# Etiology, Presentation, and Risk Factors for Diarrheal Syndromes in 3 Sub-Saharan African Countries After the Introduction of Rotavirus Vaccines From the Vaccine Impact on Diarrhea in Africa (VIDA) Study

**DOI:** 10.1093/cid/ciad022

**Published:** 2023-04-19

**Authors:** Andrea G Buchwald, Jennifer R Verani, Adama Mamby Keita, M Jahangir Hossain, Anna Roose, Samba O Sow, Richard Omore, Sanogo Doh, Joquina Chiquita M Jones, Dilruba Nasrin, Syed M A Zaman, Catherine Okoi, Martin Antonio, John B Ochieng, Jane Juma, Uma Onwuchekwa, Helen Powell, James A Platts-Mills, Sharon M Tennant, Karen L Kotloff

**Affiliations:** Center for Vaccine Development and Global Health, University of Maryland School of Medicine, Baltimore, Maryland, USA; Department of Pediatrics, University of Maryland School of Medicine, Baltimore, Maryland, USA; Division of Global Health Protection, Centers for Disease Control and Prevention, Nairobi, Kenya; Centre pour le Développement des Vaccins du Mali (CVD-Mali), Bamako, Mali; Medical Research Council Unit The Gambia at the London School of Hygiene & Tropical Medicine, Banjul, The Gambia; Center for Vaccine Development and Global Health, University of Maryland School of Medicine, Baltimore, Maryland, USA; Department of Pediatrics, University of Maryland School of Medicine, Baltimore, Maryland, USA; Centre pour le Développement des Vaccins du Mali (CVD-Mali), Bamako, Mali; Kenya Medical Research Institute, Center for Global Health Research (KEMRI-CGHR), Kisumu, Kenya; Centre pour le Développement des Vaccins du Mali (CVD-Mali), Bamako, Mali; Medical Research Council Unit The Gambia at the London School of Hygiene & Tropical Medicine, Banjul, The Gambia; Department of Pediatrics, University of Maryland School of Medicine, Baltimore, Maryland, USA; Medical Research Council Unit The Gambia at the London School of Hygiene & Tropical Medicine, Banjul, The Gambia; Medical Research Council Unit The Gambia at the London School of Hygiene & Tropical Medicine, Banjul, The Gambia; Medical Research Council Unit The Gambia at the London School of Hygiene & Tropical Medicine, Banjul, The Gambia; Kenya Medical Research Institute, Center for Global Health Research (KEMRI-CGHR), Kisumu, Kenya; Kenya Medical Research Institute, Center for Global Health Research (KEMRI-CGHR), Kisumu, Kenya; Centre pour le Développement des Vaccins du Mali (CVD-Mali), Bamako, Mali; Center for Vaccine Development and Global Health, University of Maryland School of Medicine, Baltimore, Maryland, USA; Department of Pediatrics, University of Maryland School of Medicine, Baltimore, Maryland, USA; Division of Infectious Diseases and International Health, Department of Medicine, University of Virginia, Charlottesville, Virginia, USA; Center for Vaccine Development and Global Health, University of Maryland School of Medicine, Baltimore, Maryland, USA; Department of Medicine, University of Maryland School of Medicine, Baltimore, Maryland, USA; Center for Vaccine Development and Global Health, University of Maryland School of Medicine, Baltimore, Maryland, USA; Department of Pediatrics, University of Maryland School of Medicine, Baltimore, Maryland, USA; Department of Medicine, University of Maryland School of Medicine, Baltimore, Maryland, USA

**Keywords:** diarrhea, dysentery, persistent, global, infection

## Abstract

**Background:**

Diarrheal disease is heterogeneous, including watery diarrhea (WD) and dysentery, some cases of which become persistent diarrhea (PD). Changes in risk over time necessitate updated knowledge of these syndromes in sub-Saharan Africa.

**Methods:**

The Vaccine Impact on Diarrhea in Africa (VIDA) study was an age-stratified, case-control study of moderate-to-severe diarrhea among children <5 years old in The Gambia, Mali, and Kenya (2015–2018). We analyzed cases with follow-up of about 60 days after enrollment to detect PD (lasting ≥14 days), examined the features of WD and dysentery, and examined determinants for progression to and sequelae from PD. Data were compared with those from the Global Enteric Multicenter Study (GEMS) to detect temporal changes. Etiology was assessed from stool samples using pathogen attributable fractions (AFs), and predictors were assessed using χ^2^ tests or multivariate regression, where appropriate.

**Results:**

Among 4606 children with moderate-to-severe diarrhea, 3895 (84.6%) had WD and 711 (15.4%) had dysentery. PD was more frequent among infants (11.3%) than in children 12–23 months (9.9%) or 24–59 months (7.3%), *P* = .001 and higher in Kenya (15.5%) than in The Gambia (9.3%) or Mali (4.3%), *P* < .001; the frequencies were similar among children with WD (9.7%) and those with dysentery (9.4%). Compared to children not treated with antibiotics, those who received antibiotics had a lower frequency of PD overall (7.4% vs 10.1%, *P* = .01), and particularly among those with WD (6.3% vs 10.0%; *P* = .01) but not among children with dysentery (8.5% vs 11.0%; *P* = .27). For those with watery PD, *Cryptosporidium* and norovirus had the highest AFs among infants (0.16 and 0.12, respectively), while *Shigella* had the highest AF (0.25) in older children. The odds of PD decreased significantly over time in Mali and Kenya while increasing significantly in The Gambia.

**Conclusions:**

The burden of PD endures in sub-Saharan Africa, with nearly 10% of episodes of WD and dysentery becoming persistent.

## Teaser

This report updates our knowledge of the risk factors and etiology associated with persistent diarrhea and dysentery. We demonstrate that both persistent and bloody diarrhea remain serious problems with distinct etiology, presentation, and outcomes among sub-Saharan African infants.

Diarrheal disease is the third leading cause of mortality among children <5 years of age globally, with 1 in 10 deaths attributed to diarrhea in 2019, and the greatest burden among children in South Asia and sub-Saharan Africa [1]. Although commonly described as a single entity, diarrheal disease comprises multiple clinical syndromes, each associated with different risk factors, causes, geographic distribution, pathophysiology, and sequelae. These include acute watery diarrhea (AWD) and bloody diarrhea (ie, dysentery), some cases of which progress to persistent diarrhea (PD). AWD is predominant in young children and characterized by frequent nonbloody loose or watery stools that can result in life-threatening dehydration and electrolyte abnormalities. Less common is bloody diarrhea, which historically has been associated with an increased risk of stunting [2], episodes of longer duration, and, in some settings, an increased risk of death compared with watery diarrhea (WD) [3–5]. Diarrheal episodes lasting ≥14 days, termed PD, are seen disproportionately among children in low- and middle-income countries and have been associated with more growth faltering and higher mortality rates [4, 6, 7].

During the past 3 decades, remarkable improvement has been observed in mortality rates associated with diarrheal disease in children <5 years old, attributed to declines in risks, such as unsafe water and sanitation and stunting [8], in association with social and economic development in low- and middle-income countries, coupled with improvements in case management and rotavirus vaccine introduction [9]. It is reasonable to expect that these ongoing shifts will result in changes in the etiology, manifestations, and outcomes of diarrhea in young children. However, progress has not been distributed equitably, in particular leaving areas of sub-Saharan Africa with a high prevalence of risk factors and poor outcomes [10]. Therefore, it is important to update our understanding of diarrheal diseases in sub-Saharan Africa to inform initiatives for preventing disease and death associated with diarrhea.

The Vaccine Impact on Diarrhea in Africa (VIDA) study was an age-stratified, matched case-control study that examined the incidence, etiology, and adverse clinical outcomes of moderate-to-severe diarrhea (MSD) among infants and young children after the introduction of rotavirus vaccine at 3 sites in sub-Saharan Africa. Herein we describe the features of WD and dysentery, and determinants for progression to and sequelae from PD in the VIDA study. We also examine temporal trends in PD, comparing the results with those from the Global Enteric Multicenter Study (GEMS), a similarly designed study conducted at the same sites 1 decade earlier [11, 12].

## METHODS

### Study Design and Participants

Between 11 May 2015 and 23 July 2018, children 0–59 months of age residing in 3 censused populations in sub-Saharan Africa with ongoing demographic surveillance systems (DSSs) were enrolled in VIDA [13, 14]. These sites (Basse, The Gambia; Bamako, Mali; and Siaya County, Kenya) had previously participated in GEMS between 1 December 2007 and 7 March 2011 before the introduction of the rotavirus vaccine [11, 12, 15–17]. Bansang, a demographically similar DSS area adjacent to Basse, was added in VIDA to increase the number enrolled. To permit comparisons, VIDA's clinical, epidemiological, and microbiological methods closely matched those used in GEMS. VIDA's methods [14] and the main results from GEMS and VIDA are reported elsewhere [11, 13]. Key methods are summarized below.

Enrollment in both GEMS and VIDA occurred over a 36-month period at each site. Eligible case patients were brought for care at sentinel health centers (SHC) serving the DSS population at each site for a new episode of diarrhea (≥3 abnormally loose stools within 24 hours with onset within 7 days after ≥7 diarrhea-free days), with ≥1 of the following features of MSD: sunken eyes (confirmed by the caregiver as more than normal), decreased skin turgor, intravenous hydration administered or prescribed, blood in the stool, or hospital admission recommended. We aimed to enroll the first 8–9 eligible cases per fortnight in each age stratum (infants [age 0–11 months], toddlers [12–23 months[, and children [24–59 months]). For every enrolled case patient, eligible controls were randomly selected from the site's DSS database; 1–3 diarrhea-free controls were enrolled within 2 weeks of the index case enrollment, matched for sex, residential area, and age (±2 months for children aged <12 months and ± 4 months for those aged 12–59 months) [11].

### Clinical and Epidemiological Procedures and Definitions

At enrollment, the participant’s primary caretaker underwent a standardized interview to document demographic, epidemiological, and clinical information. Each child's height/length was measured and converted to a height-for-age *z* score (HAZ) based on World Health Organization (WHO) standards [18], with HAZ <−2 considered to indicate stunting [19]. Each case patient provided a fresh stool sample to be assessed for enteropathogens. Treatment data were collected for the duration of the child's stay at the sentinel health center and for a prescription given for home treatment. A child was considered to have received antibiotics based on intent to treat (ie, administered at the SHC or a prescription was given). To estimate the duration of diarrhea, caretakers recorded daily diarrhea (presence or absence) for the ensuing 14 days onto a memory aid (Supplementary Figure 1) [12]. Degree of dehydration was categorized per WHO guidelines [20]. A modified Vesikari score was calculated based on diarrhea and vomiting duration, the maximum daily frequency of diarrheal stools and emesis episodes, fever, and the degree of dehydration (Supplementary Table 1) [21].

About 60 days after enrollment (range, 50–90 days), fieldworkers visited participants at home to assess the child's vital status and to repeat anthropometric measurements. The memory aids were reviewed with the caretaker and collected.

The diarrheal syndromes are defined as follows: (1) AWD, nonbloody MSD lasting <14 days; (2) persistent WD, WD lasting ≥14 days; and (3) bloody diarrhea (dysentery), MSD with blood in stool observed by the caretaker, clinician, or laboratory staff. Owing to a paucity of persistent bloody diarrhea cases, acute and persistent bloody diarrhea were combined for etiologic analyses.

### Laboratory Procedures

Enteropathogens were identified in whole-stool samples using a customized TaqMan Array Card that compartmentalized probe-based quantitative polymerase chain reaction (qPCR) assays [13, 17]. A qPCR cycle cutoff value <35 was considered positive. In VIDA, stool samples from all enrolled cases patients and from the first diarrhea-free control were tested. In GEMS, a random sample of case patients and their first diarrhea-free controls were tested [17].

### Statistical Methods

#### Etiology of MSD by Syndrome and Study

Pathogen attributable fractions (AFs) were calculated for the 3 sites combined, stratified by diarrheal syndrome, study (GEMS or VIDA), and age, as described elsewhere [14]. Briefly, a conditional logistic regression model was used to assign a population AF of cases to a given pathogen, adjusting for other pathogens and allowing for interactions between qPCR cycle values, diarrheal syndrome, and age group. Although GEMS originally used different analytic methods, GEMS data were reanalyzed here using the VIDA methods.

#### Risk Factors for Progression to PD and Diarrhea Duration in VIDA

Clinical, demographic, and socioeconomic predictors of PD were examined in bivariate analysis using χ^2^ tests. Among MSD case patients, clinical and demographic predictors of PD included diarrhea type, clinical findings, age, site, HAZ, and receipt of antibiotics; caregiver completion of at least primary school and electricity in the household were selected a priori as key socioeconomic predictors. The analysis for the association between prescription of antibiotics and PD focuses on antibiotics recommended by WHO for dysentery (ciprofloxacin, third-generation cephalosporins, azithromycin, and pivmecillinam) [22]. There are currently no recommendations for the use of antibiotics in the treatment of WD other than for cholera. Owing to the low number of PD episodes, further exploration of subgroup-specific associations between treatment and duration of diarrhea used the median duration of diarrhea as the outcome instead of PD. Bivariate assessment of differences between median durations by subgroup was done using Wilcoxon rank sum tests. Owing to the high prevalence of *Shigella* detection in WD identified herein and elsewhere [17], we included WD episodes in this analysis despite a lack of WHO recommendations for this indication.

We examined the odds that a diarrheal episode would become PD with increasing levels of pathogen presence (AF). Adjusted odds ratios (aORs) and 95% confidence intervals (CIs) for developing PD were calculated using multivariate logistic regression, adjusting for child age, caregiver education, the presence of electricity in the household, enrollment HAZ, and site. All other pathogens, as well as pathogen × site interaction terms, were tested for inclusion and maintained in the model if significant.

### Change in PD Over Time

We assessed whether the odds of PD among MSD cases had changed between GEMS versus VIDA, using regression models that tested for interaction between the studies and age category, study site, and diarrhea type (bloody or watery). Odds ratios and 95% CIs were reported for the probability of persistence, comparing VIDA and GEMS by age and study site. Adjusted models were constructed, including interaction terms for bloody diarrhea versus WD and site, and adjusted for stunting, antibiotic prescription, fever, vomiting, stool frequency, lethargy, dehydration, caregiver education, and household electricity.

### Clinical Outcomes

We examined the association of each syndrome with growth faltering by comparing the change in HAZ between enrollment and the 60-day follow-up visit among case patients in VIDA and their matched controls using adjusted linear regression, adjusting for the following variables selected a priori: age, study site, enrollment HAZ, caregiver education level, and follow-up time.

Few deaths were reported in the study, so to include the maximum number of participants when examining the association between diarrheal syndromes and deaths, participants without memory-aid data were included if they had a final “child health” variable (where death was recorded) and the death date could be used to infer the duration of diarrhea. Owing to low outcome numbers, adjusted models were not feasible. We used χ^2^ tests to assess the differences in frequency of deaths, comparing those who had dysentery versus WD and comparing those with PD versus no PD. Dichotomous variables were compared using χ^2^ tests, and continuous variables using Wilcoxon rank sum tests. Differences were considered statistically significant at *P* < .05.

### Ethical Review

The current study was approved by the ethical review committees at the University of Maryland, Baltimore (no. HP-00062472), the Centers for Disease Control and Prevention (reliance agreement 6729), The Gambia Government/Medical Research Council/Gambia at the London School of Hygiene & Tropical Medicine (no. 1409), the Comité d'Ethique de la Faculté de Médecine, de Pharmacie, et d'Odonto-Stomatologie, Bamako, Mali (no number), and the Kenya Medical Research Institute Scientific & Ethics Review Unit in Siaya County, Kenya (no. SSE 2996). Informed, written consent was obtained from caretakers for all participants before initiation of study procedures.

## RESULTS

### Participants and Clinical Syndromes

A total of 4606 children with MSD from the VIDA study who had pathogens assessed by means of qPCR and follow-up data are included in this analysis, with 1574, 1550, and 1482 children in The Gambia, Mali, and Kenya, respectively (Table 1). In total, 3895 case patients (84.6%) had WD, of which 376 cases (9.7%) became persistent; 711 (15.4%) had bloody diarrhea, of which 67 cases (9.4%) became persistent.

**Table 1.** Bivariate Association Between Risk Factors for Development of Persistent Diarrhea Among 4606 Children With Moderate-to-Severe Diarrhea in the Vaccine Impact on Diarrhea in Africa Study

Risk Factor	Children With MSD, No. (n = 4606)	Children With PD, No. (%) (n = 443 [9.6%])	*P* Value^a^
Demographic features			
ȃAge group			
ȃȃ0–11 m	1631	184 (11.3)	.001^a^
ȃȃ12–23 m	1618	160 (9.9)	
ȃȃ24–59 m	1357	99 (7.3)	
ȃStudy site			
ȃȃKenya	1482	229 (15.5)	<.001^a^
ȃȃThe Gambia	1574	147 (9.3)	
ȃȃMali	1550	67 (4.3)	
Socioeconomic indicators			
ȃCaretaker's educational level			
ȃȃLess than primary school	3054	255 (8.4)	<.001^a^
ȃȃAt least primary school	2303	174 (7.6)	
ȃElectricity in home			
ȃȃNo	2303	269 (11.7)	<.001^a^
ȃȃYes	2303	174 (7.6)	
Clinical findings			
ȃDiarrhea type			
ȃȃWatery	3895	376 (9.7)	.85
ȃȃBloody	711	67 (9.4)	
ȃStunting (HAZ >−2)			
ȃȃNo	3583	333 (9.3)	.16
ȃȃYes	1023	110 (10.8)	
ȃFever			
ȃȃNo	3972	381 (9.6)	.89
ȃȃYes	634	62 (9.8)	
ȃVomiting			
ȃȃNo	2463	224 (9.1)	.20
ȃȃYes	2143	219 (10.2)	
ȃ Diarrhea stools per day			
ȃȃ≤5	3804	345 (9.1)	.006^a^
ȃȃ>5	802	98 (12.2)	
ȃWHO-defined dehydration			
ȃȃNone	438	41 (9.4)	.004^a^
ȃȃSome	3527	313 (8.9)	
ȃȃSevere	641	89 (13.9)	
ȃLethargy			
ȃȃNo	3043	244 (8.0)	<.001^a^
ȃȃYes	1562	199 (12.7)	
ȃModified Vesikari score			
ȃȃSevere	1219	142 (11.7)	<.001^a^
ȃȃModerate	1845	187 (10.1)	
ȃȃMild	1538	114 (7.4)	
ȃSkin pinch slow			
ȃȃNo	3291	312 (9.5)	.62
ȃȃYes	1315	131 (10.0)	
ȃMalnutrition			
ȃȃNo	4025	386 (9.6)	.87
ȃȃYes	581	57 (9.8)	

Abbreviations: HAZ, height-for-age *z* score; MSD, moderate-to-severe diarrhea; WHO, World Health Organization.

Significant at *P* < .05 (*P* values based on χ^2^ test).

### Clinical Presentation of Diarrheal Syndromes in VIDA

**Figure 1.**
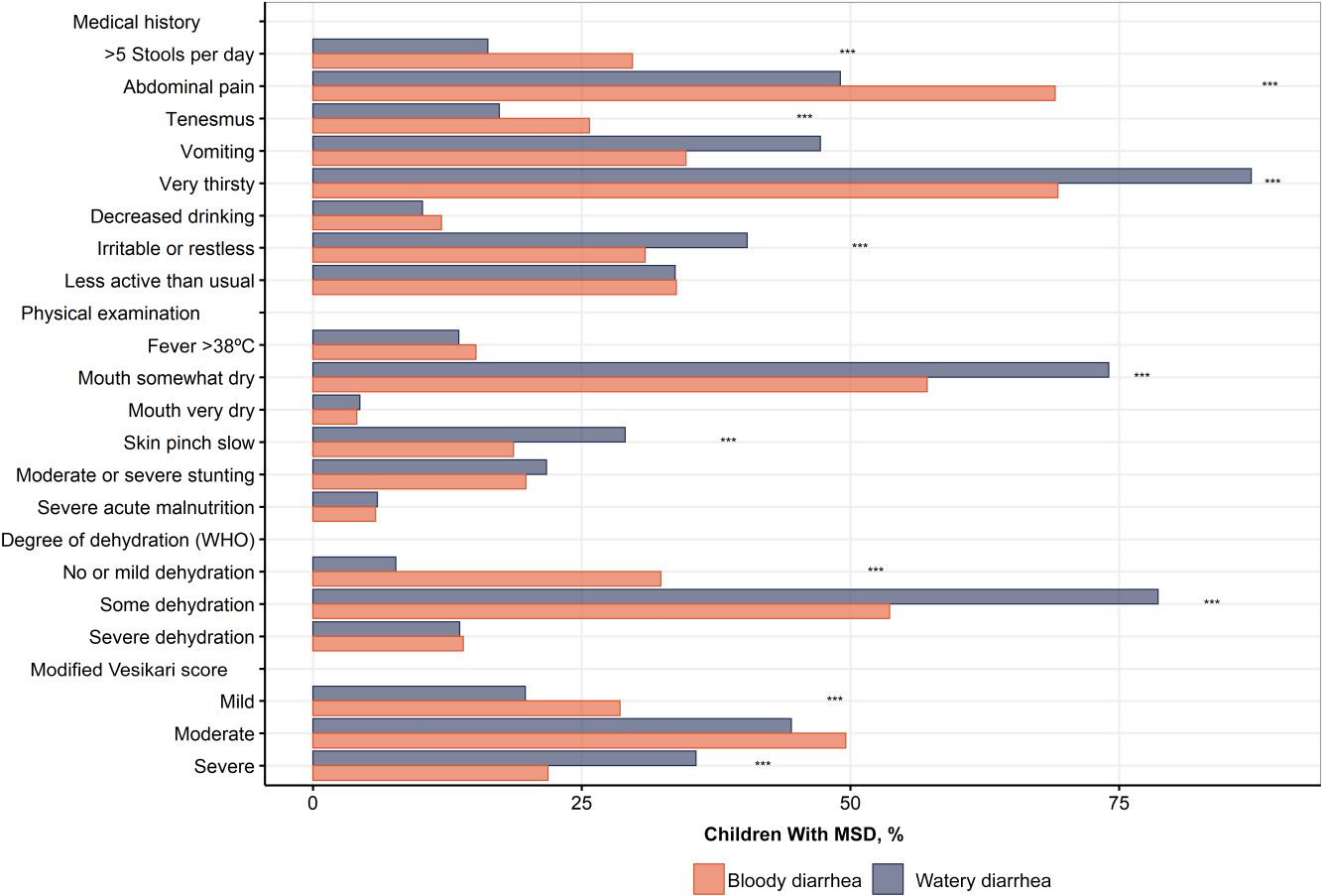
Clinical features among children with bloody or watery syndromes of moderate-to-severe diarrhea (MSD), based on findings at enrollment from medical history and physical examination. The level of severity was assessed for each syndrome at physical examination using 2 severity scores: the World Health Organization (WHO) dehydration assessment and the modified Vesikari score. ***P < .001 (χ^2^ test).

Children with bloody diarrhea were significantly more likely to pass >5 stools per day and to experience abdominal pain and tenesmus (Figure 1), while those with WD were significantly more likely to appear dehydrated, with increased thirst, decreased skin turgor, and dry mouth, which corresponded to a higher likelihood of WHO-defined dehydration and a severe modified Vesikari score.

### Etiology of MSD by Syndrome and Study

**Figure 2.**
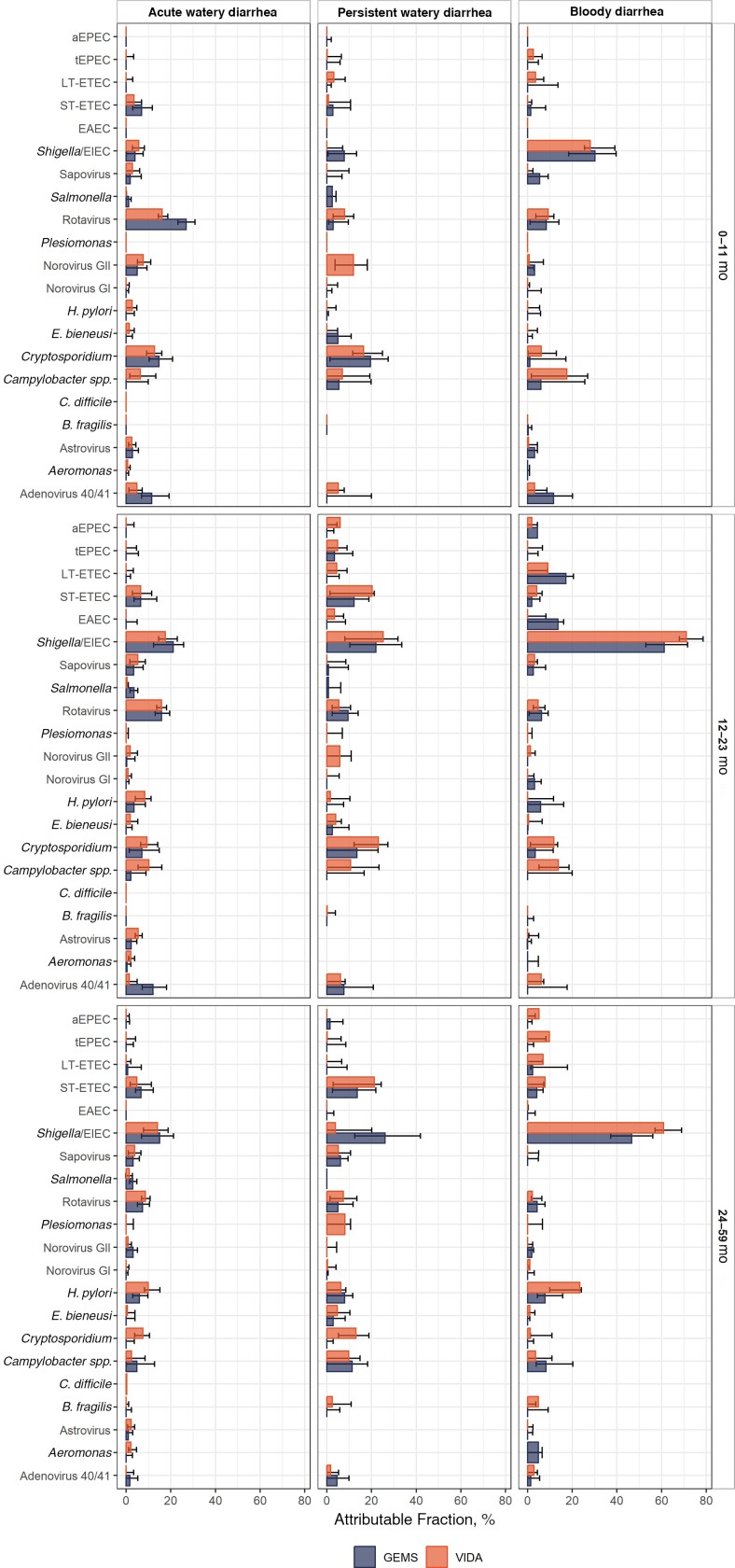
Attributable fractions and 95% confidence intervals among pathogens significantly associated with moderate-to-severe diarrhea among children residing at 3 sites in sub-Saharan Africa, by diarrhea syndrome, in the Vaccine Impact on Diarrhea in Africa (VIDA) study and the Global Enteric Multicenter Study (GEMS). Pathogen detection was performed by means of quantitative polymerase chain reaction using a customized TaqMan Array Card. *A, B,* and *C* display results for the 0–11-, 12–23-, and 24–59-month age strata, respectively. Abbreviations: aEPEC, atypical enteropathogenic *Escherichia coli*; *B. fragilis, Bacteroides fragilis*; *C. difficile, Clostridioides difficile*; *E. bieneusi, Enterocytozoon bieneusi*; EAEC, enteroaggregative *E. coli*; EIEC, enteroinvasive *E. coli*; *H. pylori, Helicobacter pylori*; LT-ETEC, heat-labile enterotoxigenic *E. coli*; ST-ETEC, heat-stable enterotoxigenic *E. coli*; tEPEC, typical enteropathogenic *E. coli*.

Pathogen AFs varied by MSD syndrome, age group, and study, although CIs overlapped (Figure 2). Among infants with AWD, rotavirus (AF, 0.16) and *Cryptosporidium* (AF, 0.13) were the pathogens most frequently classified as etiologic in VIDA. Results were similar for GEMS.

*Shigella* had the highest AFs among older children with AWD (0.17 and 0.14 for toddlers and children, respectively) in VIDA, similar to the GEMS findings. The AFs were also high for rotavirus, *Cryptosporidium, Campylobacter,* and *Helicobacter pylori* (Figure 2). *Shigella* had the highest AF among children with bloody diarrhea in all age groups in VIDA, and the AFs increased with age (0.28, 0.71, and 0.61, among children aged 0–11 months, 12-23 months, 24–59 months, respectively). *Campylobacter* had high AFs in the 2 youngest age groups (0.18 and 0.14, 0–11 months and 12–23 months, respectively). Results were similar in GEMS.

For those with persistent WD, *Cryptosporidium* (AF, 0.16) and norovirus (AF, 0.12) had the highest AFs among infants. *Shigella* (AF, 0.25), *Cryptosporidium* (AF, 0.23), and enterotoxigenic *Escherichia coli* (encoding heat-stable toxin (ST-ETEC); AF, 0.20) had the highest AFs among toddlers. ST-ETEC outranked *Shigella* in the oldest stratum (AF, 0.21), in contradistinction to GEMS. *Cryptosporidium* (AF, 0.13) and *Campylobacter* (AF, 0.10) were also prominent (Figure 2).

### Risk Factors for Progression to PD and Diarrhea Duration in VIDA

The risk of PD in VIDA was highest among infants (11.3%) and decreased with age (*P* = .001) (Table 1). The risk was significantly higher in Kenya (15.5%) and The Gambia (9.3%) than in Mali (4.3%). Lower caregiver education and lack of household electricity significantly increased the risk of PD. Children with MSD who progressed to PD were significantly more likely to present with lethargy, dehydration, and a modified Vesikari score in the severe range compared to those who did not progress to PD (Table 1).

**Figure 3.**
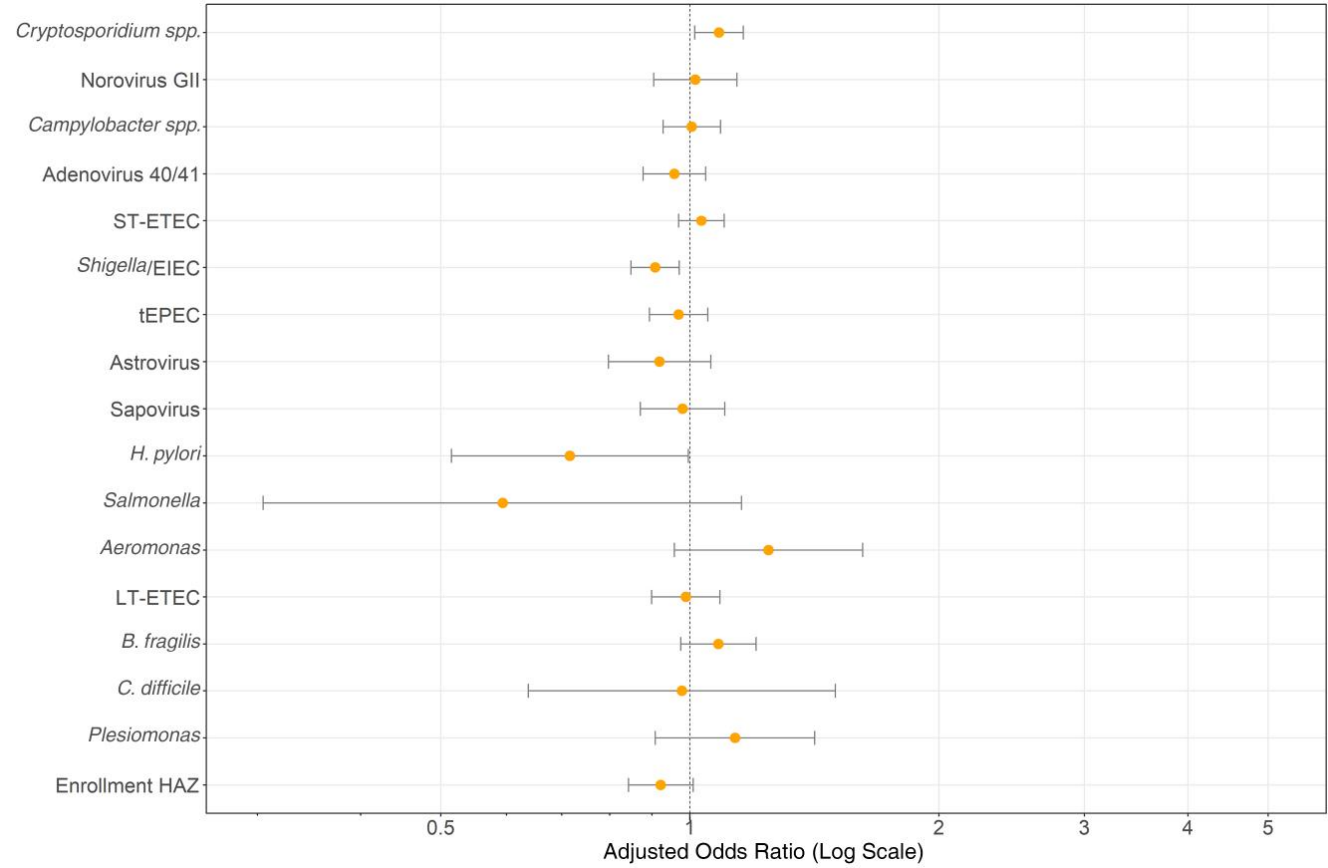
Pathogen-specific odds that an episode of moderate-to-severe diarrhea will become persistent (duration ≥14 days) among children from 3 sites in sub-Saharan Africa participating in the Vaccine Impact on Diarrhea in Africa (VIDA) study. A multivariate logistic regression model was used, combining watery and bloody diarrheal episodes, with adjustment for child age, height-for-age *z* score (HAZ) at enrollment, and study site. Results of pathogen × site interactions are shown in Supplementary Figure 2. Abbreviations: *B. fragilis, Bacteroides fragilis*; *C. difficile, Clostridioides difficile*; EIEC, enteroinvasive *Escherichia coli*; *H. pylori, Helicobacter pylori*; LT-ETEC, heat-labile enterotoxigenic *E. coli*; ST-ETEC, heat-stable enterotoxigenic *E. coli*; tEPEC, typical enteropathogenic *E. coli*.

When examined categorically, stunting at enrollment was not associated with the development of PD (Table 1). When HAZ at enrollment was examined as a continuum in multivariable regression, an increase in HAZ was not significantly associated with decreased odds of PD (aOR, 0.92 [95% CI, .84–1.01]) (Figure 3).

In multivariable regression for the association between pathogen levels (AFs) and PD, neither inclusion of caretaker education nor the presence of electricity in the household improved model fits, so these factors were not included in the final models. *Cryptosporidium* was the only pathogen significantly associated with an increased aOR for PD after adjustment for age and site. rotavirus, *H. pylori,* and *Shigella* were associated with decreased odds of PD (Figure 3 and Supplementary Figure 2).

### Impact of Treatment on PD and Duration of Diarrhea in VIDA by Clinical Syndrome

Among 4560 case patients with available data, 424 of 697 (62.3%) with dysentery and 448 of 3863 (11.6%) with WD were prescribed WHO-recommended antibiotics (Supplemental Table 3). PD developed less often in children who were prescribed antibiotics (7.4%) than in those who were not (10.1%) overall (*P* = .01), and among those with WD (6.3% vs 10.0%; *P* = .01) but not among children with dysentery (8.5% vs 11.0%; *P* = .27) (Table 2). However, when we examined the impact of treatment on the linear duration of bloody diarrhea, a significant shortening was observed (*P* = .001) (Table 3).

**Table 2.** Bivariate Association Between Prescription of World Health Organization–Recommended Antibiotics and Development of Persistent Diarrhea (≥14-Day Duration)

	Children With MSD, No. (%)^a^			
Diarrhea Type by Antibiotic Prescription Status	Total	Not Persistent	Persistent	*P* Value
**Any diarrhea**	n = 4560	n = 4124	n = 436	
ȃNo antibiotics prescribed	3678 (80.4)	3307 (80.2)	371 (85.1)	.01^b^
ȃAntibiotics prescribed	882 (19.6)	817 (19.8)	65 (14.9)	
**Acute bloody diarrhea**	n = 697	n = 631	n = 66	
ȃNo antibiotics prescribed	263 (37.7)	234 (37.1)	29 (43.9)	.27
ȃAntibiotics prescribed	434 (62.3)	397 (62.9)	37 (56.1)	
**Acute watery diarrhea**	n = 3863	n = 3493	n = 370	
ȃNo antibiotics prescribed	3415 (88.4)	3073 (88)	342 (92.4)	.01^b^
ȃAntibiotics prescribed	448 (11.6)	420 (12)	28 (7.6)	

Abbreviation: MSD, moderate-to-severe diarrhea.

Including all children with MSD for whom prescribing information was available.

Significant at *P* < .05 (*P* values based on χ^2^ test).

**Table 3.** Association Between Treatment With World Health Organization—Recommended Antibiotics and Duration of Diarrhea in Children With Moderate-to-Severe Diarrhea From the Vaccine Impact on Diarrhea in Africa Study, Stratified by Enrollment Characteristics

Characteristic by Diarrhea Type at Enrollment	Duration of Diarrhea, Median (IQR), d		
	No Antibiotic Prescribed (n = 3678)	Antibiotic Prescribed (n = 882)	*P* Value
Bloody diarrhea	6 (4–9)	5 (4–8)	**.001** ^a^
ȃAge group			
ȃȃ0–11 m	7 (5–11)	6 (4–10)	.15
ȃȃ12–23 m	7 (5–9)	5 (4–8)	**.002** ^a^
ȃȃ24–59 m	5 (3–9)	5 (3–7.5)	.56
ȃSite			
ȃȃKenya	7 (4–11)	5 (4–11)	.28
ȃȃMali	6 (5–8)	4 (3–7)	.22
ȃȃThe Gambia	6 (4–9)	5 (4–8)	.19
ȃ*Shigella* detected			
ȃȃNo	6 (5–11)	6 (4–10)	.09
ȃȃYes	6 (4–9)	5 (3–8)	**.03** ^a^
ȃStunting (HAZ >−2)			
ȃȃYes	7 (5–11)	5 (3–10)	**.02** ^a^
ȃȃNo	6 (4–9)	5 (4–8)	**.02** ^a^
Watery diarrhea	5 (4–8)	5 (3–7)	.06
ȃAge group			
ȃȃ0–11 m	6 (4–8)	6 (4–8)	.89
ȃȃ12–23 m	5 (4–8)	5 (3–8)	**.05** ^a^
ȃȃ24–59 m	5 (3–7)	4 (3–6)	.10
ȃSite			
ȃȃKenya	6 (4–10)	7.5 (4–9)	.86
ȃȃMali	4 (3–6)	5 (3–7)	.29
ȃȃThe Gambia	5 (4–9)	5 (3–8)	**.03** ^a^
ȃ*Shigella* detected			
ȃȃNo	5 (4–8)	5 (4–7)	.57
ȃȃYes	5 (4–8)	5 (3–7)	**.02** ^a^
ȃStunting (HAZ >−2)			
ȃȃYes	5 (4–9)	5 (4–8)	.30
ȃȃNo	5 (4–8)	5 (3–7)	.12

Abbreviations: HAZ, height-for-age *z* score; IQR, interquartile range.

Significant at *P* < .05 ( *P* values based on Wilcoxon rank sum test).

In subgroup analyses, treatment was significantly associated with shorter diarrhea duration among toddlers 12–23 months of age (*P* = .002) and among children with *Shigella* detection (*P* = .03). The effect was comparable in children with and those without stunting (*P* = .02 for both). A similar trend was seen for WD (*P* = .06), where a significant decrease in diarrhea duration associated with treatment was also seen in The Gambia (*P* = .03) and in children with shigellosis (*P* = .02).

Overall, zinc was prescribed in VIDA in 96.7% of MSD cases in Kenya, 5.7% in Mali, and 48.5% in The Gambia (Supplementary Table 4). There was no association between zinc and PD in Kenya and Mali, likely owing to the homogeneity of zinc prescribing. In The Gambia, zinc was associated with a decreased risk of PD (*P* = .01).

### Change in Odds of PD Between GEMS and VIDA

By comparison, 12.2% of the 4535 GEMS cases of WD became persistent, along with 14.7% of the 565 bloody diarrhea cases (Supplementary Table 2). Compared with GEMS, in the VIDA study the odds of MSD persisting decreased significantly in Mali and Kenya for both WD and bloody diarrhea (Table 4). These trends did not vary significantly by age group and remained after adjustment for other factors. By contrast, in The Gambia, the odds of WD becoming persistent were >2-fold higher in VIDA compared with GEMS; this risk was observed in all age groups and after adjustment for other factors. The odds of bloody diarrhea becoming persistent were also higher in The Gambia in VIDA compared with GEMS. However, this increase was not statistically significant overall or in any age group. The adjustment did not change this relationship.

**Table 4.** Odds of Persistent Diarrhea and Adjusted Odds Ratio for Persistent Diarrhea Comparing the Vaccine Impact on Diarrhea in Africa Study With the Global Enteric Multicenter Study

Diarrhea Type by Location and Study	Odds of PD	aOR (95% CI)^a^
Watery diarrhea		
ȃThe Gambia		
ȃȃGEMS	0.06	Reference
ȃȃVIDA	0.11	2.2 (1.6–3.2)
ȃMali		
ȃȃGEMS	0.11	Reference
ȃȃVIDA	0.04	0.4 (.3–.5)
ȃKenya		
ȃȃGEMS	0.25	Reference
ȃȃVIDA	0.19	0.8 (.6–.9)
Bloody diarrhea		
ȃThe Gambia		
ȃȃGEMS	0.06	Reference
ȃȃVIDA	0.09	1.5 (.9–2.3)
ȃMali		
ȃȃGEMS	0.15	Reference
ȃȃVIDA	0.09	0.3 (.2–.4)
ȃKenya		
ȃȃGEMS	0.39	Reference
ȃȃVIDA	0.15	0.5 (.3–.7)

Abbreviations: aOR, adjusted odds ratio; CI, confidence interval; GEMS, Global Enteric Multicenter Study; PD, persistent diarrhea; VIDA, Vaccine Impact on Diarrhea in Africa.

Adjusted using logistic regression for odds of PD in VIDA versus GEMS, including interaction terms for bloody versus watery diarrhea and site and adjustment for stunting, antibiotic prescription, fever, vomiting, stool frequency, lethargy, dehydration, caregiver educational level, and household electricity.

### Clinical Outcomes

We examined the association of each syndrome with growth faltering by comparing the change in HAZ between enrollment and the 60-day follow-up visit among case patients in VIDA and their matched controls (Table 5). Compared with controls, all cases had more growth faltering, except those with persistent bloody diarrhea. There was no association between PD and growth faltering when comparing AWD with watery PD. Thirty-seven MSD case patients in VIDA died. There was no significant difference in the frequency of death comparing children with bloody diarrhea and those with WD (5 of 722 [0.69%] vs 32 of 3934 [0.81%], respectively; *P* = .74). Among the those who died, 35 had sufficient duration data, and the proportion who died was higher among those with than among with those without PD (6 of 440 [1.36%] vs 29 of 4214 [0.69%], respectively; *P* = .12), but this difference was not significant.

**Table 5.** Association Between Change in Height-for-Age *z* Score and Diarrheal Syndrome From Adjusted Linear Regression Model

Characteristic	Change in HAZ Compared With Reference Group	Standard Error	*P* Value
Diarrheal syndrome			
**ȃ**Controls	Reference	…	…
**ȃ**Case patients			
**ȃȃ**Acute bloody diarrhea	−0.036	0.015	.02
**ȃȃ**Acute watery diarrhea	−0.076	0.008	<.001
**ȃȃ**Persistent bloody diarrhea	0.060	0.043	.16
**ȃȃ**Persistent watery diarrhea	−0.076	0.019	<.001
Age group			
**ȃ**0–11 m	Reference	…	…
**ȃ**12–23 m	0.136	0.009	<.001
**ȃ**24–59 m	0.231	0.009	<.001
Study site			
**ȃ**The Gambia	Reference	…	…
**ȃ**Kenya	0.054	0.010	<.001
**ȃ**Mali	0.084	0.009	<.001
Enrollment HAZ^a^	−0.067	0.003	<.001
Caregiver educational level			
**ȃ**Less than primary school	Reference	…	…
**ȃ**At least primary school	0.030	0.009	<.001

Abbreviation: HAZ, height-for-age *z* score.

Adjusted for age, site, enrollment HAZ, caregiver education level, and follow-up time.

Values for enrollment HAZ indicate the effect of enrollment HAZ on change in HAZ.

## DISCUSSION

Our findings indicate that PD continues to be a public health problem in sub-Saharan Africa, though the proportion of diarrheal episodes progressing to PD has decreased in the decade between GEMS and VIDA in Mali and Kenya but not in The Gambia [23, 24]. Among episodes of medically attended MSD, we found that PD developed in nearly 1 in 10 children. Those with persistent WD not only had significantly more growth faltering compared with controls, but they also had a higher probability of dying within 2–3 months of enrollment than those with AWD, although this difference was not significant.

Site-to-site differences in the proportion of MSD episodes that proceed to PD were apparent, ranging from 4.3% in Mali to 15.5% in Kenya. In addition, after introduction of rotavirus vaccination, the proportional distribution of PD increased in The Gambia but decreased significantly in both Mali and Kenya. Because we used consistent methods across our sites, we suspect that the patterns reflect inherent differences in exposure to factors that may affect diarrhea duration, such as malnutrition [25]), improved sanitation and hygiene [26], treatment with antibiotics and zinc [27], reduced human immunodeficiency virus exposure [28], and AF of etiologic agents.

We found that the risk of PD was higher among infants 0–11 months of age than in older children, as others have reported [7, 29–33]. This pattern coincides with the peak incidence of acute diarrhea and has been attributed to increased susceptibility to enteric infections among immunologically naive infants, perhaps in concert with other age-related vulnerabilities [31, 33–35]. In our study, as in GEMS, the clinical severity of the presenting illness increased the likelihood of progression to PD. Similar findings have been reported elsewhere [32, 36, 37], although the impact of dehydration as a predisposing condition has been inconsistent [29]. Notably, PD was associated with severe but not acute dehydration, which raises the possibility that clinical signs we observed, such as lethargy and decreased intake, which meet WHO criteria for severe dehydration, may actually have been attributable to an alternative disease process.

In contrast to other reports [3–5], the likelihood of progressing to PD was not greater for bloody diarrhea than for WD. On the other hand, sociodemographic factors such as lack of maternal primary education and indicators of low household wealth have consistently been found as risk factors in our study and others [31, 34, 38]. The presence of rotavirus was associated with decreased odds of progression to PD, which may have been related to the characteristically short lived rotavirus infection.

Defining whether malnutrition is a risk factor for the development of PD has proved to be challenging [39]. We did not observe an association between stunting as a categorical variable and PD, but we did observe a trend suggesting an association between lower enrollment HAZ and PD.

We estimated the population AF of enteropathogens present early in the illness and significantly associated with each diarrheal syndrome controlling for the presence of other pathogens. Rotavirus remained a significant cause of AWD among children <24 months of age, despite the introduction of rotavirus vaccination between GEMS and VIDA at all sites. *Shigella* was the most important pathogen among cases of bloody diarrhea in all age groups in both GEMS and VIDA. *Campylobacter* spp. was also associated with bloody diarrhea, as reported elsewhere, having increased among children <24 months of age in VIDA compared with GEMS [3, 40–42]. In VIDA, the large AF for *H. pylori* among episodes of both bloody diarrhea and AWD among children 24–59 months of age was unexpected, although similar observations were seen in GEMS and remain of uncertain significance [43].

The AF of *Cryptosporidium* was comparable to rotavirus among infants with AWD and was a leading cause of persistent WD at all ages, as reported elsewhere [31, 44, 45]. Although not significant in multivariable analysis, we and others found additional pathogens that were associated with PD in bivariate analysis, including ST-ETEC [31, 39], *Shigella* [39, 46], norovirus, and *Campylobacter* spp. [46]. Norovirus was associated with persistent WD in VIDA but not in GEMS [11].

Antibiotics appeared to confer a significant reduction in the duration of both bloody diarrhea and WD that seemed to be driven by the impact on shigellosis. This presents a dilemma, since the risk of PD among children with WD (9.7%), for which no recommendation for the use of antibiotics exists, is comparable to that among children with bloody diarrhea (9.4%), which has a treatment indication. Although our findings provide an argument for expanded treatment of diarrheal diseases in sub-Saharan Africa, the risk of emerging antibiotic resistance remains problematic. Regardless, innovative nutritional rehabilitation [47], accelerated development of *Shigella* vaccines [48–50], and enhanced messaging to encourage the use of zinc for diarrheal diseases is warranted.

In this population, children with persistent bloody diarrhea were no more likely to have growth faltering than controls. Those with bloody diarrhea had a high rate (62.4%) of treatment with antibiotics, and our group has previously demonstrated that that children with *Shigella* who receive antibiotics grow better [51]. Thus, the lack of association between bloody diarrhea and growth faltering may well be the result of antibiotic use.

The current study had several strengths. While community-based studies are ideal for studying risk factors of PD, there have been few since the 1990s [35], or in Africa [34, 38]. VIDA's design optimized our ability to assess risk factors and etiology by collecting data within 7 days of episode onset and analyzing linked specimens using highly sensitive qPCR tests. Longitudinal follow-up of case patients and controls using a memory aid allowed prospective detection of episodes that became PD. We were able to compare our findings from VIDA and GEMS, a study conducted 10 years earlier using the same study sites and nearly identical methods to assess temporal changes.

Nonetheless, this study has several limitations. The definition of PD relied on the accurate completion of our memory aid by caretakers with high illiteracy levels. Fortunately, compliance was high, and data integrity was optimized using training and the involvement of literate family members. Our study was not designed to assess compliance with antibiotic or zinc treatment, so we performed an “intent-to-treat” analysis. It is possible that the PD cases included may not be generalizable to PD episodes that do not result in medical care. Attribution of a diarrheal episode to an individual pathogen is difficult, as many cases had multiple pathogens detected, and qPCR cannot ensure the presence of a clinically significant infection. However, our ability to compare case patients with age- and site-matched controls improves the accuracy of identification of likely etiologic agents. Finally, owing to the small number of deaths in the study, we were not able to adjust for likely confounders, and our power to detect an association was limited.

In conclusion, our findings demonstrate that the burden of diarrheal disease continues in sub-Saharan Africa, with nearly 10% of episodes of WD and bloody diarrhea becoming persistent. After several decades in which research on diarrheal syndromes have paused, we have characterized clinical presentations, sociodemographic risk factors, and etiologic agents that can help build a contemporary knowledge base to inform interventions and case management strategies.

## Supplementary Data

Supplementary materials are available at *Clinical Infectious Diseases* online. Consisting of data provided by the authors to benefit the reader, the posted materials are not copyedited and are the sole responsibility of the authors, so questions or comments should be addressed to the corresponding author.

## Supplementary Material

ciad022_Supplementary_Data

## Notes

***Acknowledgments.*** The authors express their deep gratitude to the families who participated in these studies, the clinical and field staff for their exceptional hard work and dedication, and the physicians, administration, and health officials at every site who generously provided facilities and support for the conduct of the study. They are grateful to Catherine Johnson, Chris Focht, and Nora Watson at the Emmes Company for expert data management and reporting. Special thanks go to Carl Kirkwood, Duncan Steele, and Anita Zaidi at the Bill & Melinda Gates Foundation for helpful oversight, Kathy Neuzil for thoughtful suggestions, and to the following members of the authors' International Scientific Advisory Committee for providing insightful comments and guidance: Janet Wittes (chair), George Armah, John Clemens, Christopher Duggan, Stephane Helleringer, Ali Mokdad, James Nataro, and Halvor Sommerfelt.

***Disclaimer.*** The funding organizations had no role in the design, collection, analysis, or interpretation of data, or in the writing of the manuscript. The findings and conclusions in this report are those of the authors and do not necessarily represent the official position of the Kenya Medical Research Institute, the University of Maryland, the US Centers for Disease Control and Prevention, or any of the collaborating partners in this project.

***Financial support.*** This work was supported by the Bill & Melinda Gates Foundation (grants OPP1111236 and OPP1116751).

***Supplement sponsorship.*** This supplement was sponsored by the Bill & Melinda Gates Foundation. This study is based on research funded in part by grants from the Bill & Melinda Gates Foundation (OPP1111236/OPP1116751).

## References

[1] Paulson KR , Kamath AM, Alam T, et al Global, regional, and national progress towards Sustainable Development Goal 3.2 for neonatal and child health: all-cause and cause-specific mortality findings from the Global Burden of Disease Study 2019. Lancet 2021; 398:870–905. 34416195 10.1016/S0140-6736(21)01207-1 PMC8429803

[2] Briend A , Aziz K, Hasan KZ, Hoque B. Are diarrhoea control programmes likely to reduce childhood malnutrition? Observations from rural Bangladesh. Lancet 1989; 334:319–22. 10.1016/s0140-6736(89)90498-4 2569114

[3] Kuşkonmaz B , Yurdakök K, Yalcin S, Ozmert E. Comparison of acute bloody and watery diarrhea: a case control study. Turk J Pediatr 2009; 51:133–40. 19480324

[4] Persistent diarrhoea in children in developing countries: memorandum from a WHO meeting. Bull World Health Organ 1988; 66:709–17. 3266111 PMC2491148

[5] Ronsmans C , Bennish M, Wierzba T. Diagnosis and management of dysentery by community health workers. Lancet 1988; 332:552–5. 10.1016/s0140-6736(88)92669-4 2900931

[6] Bhandari N , Bhan M, Sazawal S. Mortality associated with acute watery diarrhea, dysentery and persistent diarrhea in rural north India. Acta Paediatr 1992; 81:3–6. 12286021

[7] Moore SR , Lima NL, Soares AM, et al Prolonged episodes of acute diarrhea reduce growth and increase risk of persistent diarrhea in children. Gastroenterology 2010; 139:1156–64. 20638937 10.1053/j.gastro.2010.05.076 PMC2949449

[8] Murray CJ , Aravkin AY, Zheng P, et al Global burden of 87 risk factors in 204 countries and territories, 1990–2019: a systematic analysis for the Global Burden of Disease Study 2019. Lancet 2020; 396:1223–49. 33069327 10.1016/S0140-6736(20)30752-2 PMC7566194

[9] Black R , Fontaine O, Lamberti L, et al Drivers of the reduction in childhood diarrhea mortality 1980–2015 and interventions to eliminate preventable diarrhea deaths by 2030. J Glob Health 2019; 9:020801. 10.7189/jogh.09.020801 PMC6815873 31673345

[10] Olofin I , McDonald CM, Ezzati M, et al Associations of suboptimal growth with all-cause and cause-specific mortality in children under five years: a pooled analysis of ten prospective studies. PLoS One 2013; 8:e64636. 10.1371/journal.pone.0064636 PMC3667136 23734210

[11] Kotloff KL , Nataro JP, Blackwelder WC, et al Burden and aetiology of diarrhoeal disease in infants and young children in developing countries (the Global Enteric Multicenter Study, GEMS): a prospective, case-control study. Lancet 2013; 382:209–22. 23680352 10.1016/S0140-6736(13)60844-2

[12] Kotloff KL , Blackwelder WC, Nasrin D, et al The Global Enteric Multicenter Study (GEMS) of diarrheal disease in infants and young children in developing countries: epidemiologic and clinical methods of the case/control study. Clin Infect Dis 2012; 55(suppl 4):S232–45. 23169936 10.1093/cid/cis753 PMC3502307

[13] Kotloff KL , Sow SO, Hossain MJ, et al Changing landscape of moderate-to-severe diarrhea among children in 3 sub-Saharan African countries following rotavirus vaccine introduction: the Vaccine Impact on Diarrhea in Africa (VIDA). In preparation.

[14] Powell H , Liang Y, Neuzil KM, et al A description of the statistical methods for the Vaccine Impact on Diarrhea in Africa (VIDA) study. Clin Infect Dis 2023; 76(Suppl 1):S5–11. 37074428 10.1093/cid/ciac968 PMC10116558

[15] Panchalingam S , Antonio M, Hossain A, et al Diagnostic microbiologic methods in the GEMS-1 case/control study. Clin Infect Dis 2012; 55(suppl 4):S294–302. 23169941 10.1093/cid/cis754 PMC3502308

[16] Blackwelder WC , Biswas K, Wu Y, et al Statistical methods in the Global Enteric Multicenter Study (GEMS). Clin Infect Dis 2012; 55(suppl 4):S246–53. 23169937 10.1093/cid/cis788 PMC3502316

[17] Liu J , Platts-Mills JA, Juma J, et al Use of quantitative molecular diagnostic methods to identify causes of diarrhoea in children: a reanalysis of the GEMS case-control study. Lancet 2016; 388:1291–301. 27673470 10.1016/S0140-6736(16)31529-X PMC5471845

[18] WHO Multicentre Growth Reference Study Group . WHO child growth standards: length/height-for-age, weight-for-age, weight-for-length, weight-for-height and body mass index-for-age: methods and development.World Health Organization,**2006**.

[19] World Health Organization . Reducing stunting in children: equity considerations for achieving the global nutrition targets 2025. Geneva, Switzerland: World Health Organization, 2018.

[20] World Health Organization . Handbook: IMCI integrated management of childhood illness. Available at: https://pdf.usaid.gov/pdf_docs/pnadg515.pdf. Accessed 22 March 2012.

[21] Ruuska T , Vesikari T. Rotavirus disease in Finnish children: use of numerical scores for clinical severity of diarrhoeal episodes. Scand J Infect Dis 1990; 22:259–67. 2371542 10.3109/00365549009027046

[22] World Health Organization . Guidelines for the control of shigellosis, including epidemics due to Shigella dysenteriae type 1. Geneva, Switzerland: World Health Organization, 2005.

[23] Das SK , Faruque AS, Chisti MJ, Malek MA, Salam MA, Sack DA. Changing trend of persistent diarrhoea in young children over two decades: observations from a large diarrhoeal disease hospital in Bangladesh. Acta Paediatr 2012; 101:e452–7. 22734659 10.1111/j.1651-2227.2012.02761.x

[24] Schorling JB , Wanke CA, Schorling SK, McAuliffe JF, de Souza MA, Guerrant RL. A prospective study of persistent diarrhea among children in an urban Brazilian slum. Patterns of occurrence and etiologic agents. Am J Epidemiol 1990; 132:144–56. 2192547 10.1093/oxfordjournals.aje.a115626

[25] Schorling JB , McAuliffe JF, de Souza MA, Guerrant RL. Malnutrition is associated with increased diarrhoea incidence and duration among children in an urban Brazilian slum. Int J Epidemiol 1990; 19:728–35. 2262271 10.1093/ije/19.3.728

[26] Guerrant DI , Moore SR, Lima AA, Patrick PD, Schorling JB, Guerrant RL. Association of early childhood diarrhea and cryptosporidiosis with impaired physical fitness and cognitive function four–seven years later in a poor urban community in northeast Brazil. Am J Trop Med Hyg 1999; 61:707–13. 10586898 10.4269/ajtmh.1999.61.707

[27] Patel A , Mamtani M, Dibley MJ, Badhoniya N, Kulkarni H. Therapeutic value of zinc supplementation in acute and persistent diarrhea: a systematic review. PLoS One 2010; 5:e10386. 10.1371/journal.pone.0010386 PMC2860998 20442848

[28] Muttai H , Guyah B, Achia T, et al Mapping geographic clusters of new HIV diagnoses to inform granular-level interventions for HIV epidemic control in western Kenya. BMC Public Health 2021; 21:1926. 34688267 10.1186/s12889-021-11890-7 PMC8542332

[29] Strand TA , Sharma PR, Gjessing HK, et al Risk factors for extended duration of acute diarrhea in young children. PLoS One 2012; 7:e36436. 10.1371/journal.pone.0036436 PMC3348155 22590543

[30] Black RE . Persistent diarrhea in children of developing countries. Pediatr Infect Dis J 1993; 12:751–61; discussion 62-4. 8414804 10.1097/00006454-199309000-00010

[31] Lima AA , Moore SR, Barboza MS Jr, et al Persistent diarrhea signals a critical period of increased diarrhea burdens and nutritional shortfalls: a prospective cohort study among children in northeastern Brazil. J Infect Dis 2000; 181: 1643–51. 10823764 10.1086/315423

[32] Baqui AH , Black RE, Sack RB, Yunus MD, Siddique AK, Chowdhury HR. Epidemiological and clinical characteristics of acute and persistent diarrhoea in rural Bangladeshi children. Acta Paediatr Suppl 1992; 381:15–21. 1421934 10.1111/j.1651-2227.1992.tb12366.x

[33] Karim A , Akhter S, Rahman M, Nazir M. Risk factors of persistent diarrhea in children below five years of age. Indian J Gastroenterol 2001; 20:59–61. 11305492

[34] Mølbak K , Jensen H, Ingholt L, Aaby P. Risk factors for diarrheal disease incidence in early childhood: a community cohort study from Guinea-Bissau. Am J Epidemiol 1997; 146:273–82. 9247012 10.1093/oxfordjournals.aje.a009263

[35] Bhutta ZA , Nelson EA, Lee WS, et al Recent advances and evidence gaps in persistent diarrhea. J Pediatr Gastroenterol Nutr 2008; 47:260–5. 18664885 10.1097/MPG.0b013e318181b334

[36] Lanata CF , Black RE, Gilman RH, Lazo F, Del Aguila R. Epidemiologic, clinical, and laboratory characteristics of acute vs. persistent diarrhea in periurban Lima, Peru. J Pediatr Gastroenterol Nutr 1991; 12:82–8. 2061784 10.1097/00005176-199101000-00017

[37] Patel AB , Ovung R, Badhoniya NB, Dibley MJ. Risk factors for predicting diarrheal duration and morbidity in children with acute diarrhea. Indian J Pediatrics 2012; 79:472–7. 10.1007/s12098-011-0561-3 21948223

[38] Schilling KA , Omore R, Derado G, et al Factors associated with the duration of moderate-to-severe diarrhea among children in rural Western Kenya enrolled in the Global Enteric Multicenter Study, 2008–2012. Am J Trop Med Hygiene 2017; 97:248–58. 10.4269/ajtmh.16-0898 PMC5508904 28719331

[39] Black RE , Brown KH, Becker S. Effects of diarrhea associated with specific enteropathogens on the growth of children in rural Bangladesh. Pediatrics 1984; 73:799–805. 6374599

[40] Njuguna C , Njeru I, Mgamb E, et al Enteric pathogens and factors associated with acute bloody diarrhoea, Kenya. BMC Infect Dis 2016; 16:1–10. 27600526 10.1186/s12879-016-1814-6 PMC5012060

[41] Brooks JT , Shapiro RL, Kumar L, et al Epidemiology of sporadic bloody diarrhea in rural Western Kenya. Am J Trop Med Hygiene 2003; 68:671–7. 12892051

[42] Townes JM , Quick R, Gonzales OY, et al Etiology of bloody diarrhea in Bolivian children: implications for empiric therapy. J Infect Dis 1997; 175:1527–30. 9180200 10.1086/516493

[43] Kotloff KL , Nasrin D, Blackwelder WC, et al The incidence, aetiology, and adverse clinical consequences of less severe diarrhoeal episodes among infants and children residing in low-income and middle-income countries: a 12-month case-control study as a follow-on to the Global Enteric Multicenter Study (GEMS). Lancet Global Health 2019; 7:e568–84. 31000128 10.1016/S2214-109X(19)30076-2 PMC6484777

[44] DuPont HL . Persistent diarrhea: a clinical review. JAMA 2016; 315:2712–23. 27357241 10.1001/jama.2016.7833

[45] Sodemann M , Jakobsen MS, Mølbak K, Martins C, Aaby P. Episode-specific risk factors for progression of acute diarrhoea to persistent diarrhoea in west African children. Trans R Soc Trop Med Hyg 1999; 93:65–8. 10.1016/s0035-9203(99)90183-9 10492794

[46] Islam SB , Ahmed T, Mahfuz M, et al The management of persistent diarrhoea at Dhaka Hospital of the International Centre for Diarrhoeal Disease and Research: a clinical chart review. Paediatr Int Child Health 2018; 38:87–96. 28475437 10.1080/20469047.2017.1315911

[47] Mostafa I , Fahim SM, Das S, et al Developing shelf-stable microbiota directed complementary food (MDCF) prototypes for malnourished children: study protocol for a randomized, single-blinded, clinical study. BMC Pediatr 2022; 22:385. 35778675 10.1186/s12887-022-03436-6 PMC9247958

[48] Phalipon A , Mulard LA. Toward a multivalent synthetic oligosaccharide-based conjugate vaccine against *Shigella*: state-of-the-art for a monovalent prototype and challenges. Vaccines (Basel) 2022; 10:403. 35335035 10.3390/vaccines10030403 PMC8954881

[49] Talaat KR , Alaimo C, Martin P, et al Human challenge study with a *Shigella* bioconjugate vaccine: analyses of clinical efficacy and correlate of protection. EBioMedicine 2021; 66:103310. 10.1016/j.ebiom.2021.103310 PMC8054157 33862589

[50] Barry EM , Levine MM. A tale of two bacterial enteropathogens and one multivalent vaccine. Cell Microbiol 2019; 21:e13067. 10.1111/cmi.13067 PMC6842089 31194282

[51] Nasrin D , Blackwelder WC, Sommerfelt H, et al Pathogens associated with linear growth faltering in children with diarrhea and impact of antibiotic treatment: the Global Enteric Multicenter Study. J Infect Dis 2021; 224(suppl 7):S848–55. 34528677 10.1093/infdis/jiab434 PMC8958895

