# Supplementary material for: Etiology, Presentation, and Risk Factors for Diarrheal Syndromes in 3 Sub-Saharan African Countries After the Introduction of Rotavirus Vaccines From the Vaccine Impact on Diarrhea in Africa (VIDA) Study
Source: Clin Infect Dis. 2023 Apr 19;76(Suppl 1):S12–22. doi: 10.1093/cid/ciad022 (PMC10116565; doi:10.1093/cid/ciad022)
Supplement: ciad022_Supplementary_Data [file ciad022_supplementary_data.docx]

**SUPPLEMENTAL MATERIAL**

Etiology, presentation, and risk factors for diarrheal syndromes in three sub-Saharan African countries after the introduction of rotavirus vaccines from the VIDA study

Andrea G. Buchwald, Jennifer R. Verani, Adama Mamby Keita, M. Jahangir Hossain, Anna Roose, Samba O. Sow, Richard Omore, Sanogo Doh, Joquina Chiquita M. Jones, Dilruba Nasrin, Syed M.A. Zaman, Catherine Okoi, Martin Antonio, John B. Ochieng, Jane Juma, Uma Onwuchekwa, Helen Powell, James A. Platts-Mills, Sharon M. Tennant, Karen L. Kotloff

| **Table 1. Comparison of Vesikari Score and the modified Vesikari Score (mVS) used in VIDA** | | | | | | |
| --- | --- | --- | --- | --- | --- | --- |
| **Parameter** | **Score** | | | | | **Comment** |
|  | **Vesikari** | | | | |  |
|  | **1** | **2** | | | **3** |  |
| Diarrhea |  |  | | |  |  |
| Max. no. stools/day | 1-3 | 4-5 | | | >6 |  |
| Duration (days) | 1-4 | 5 | | | >6 |  |
| Vomiting |  |  | | |  |  |
| Max. no. emesis/day | 1 | 2-4 | | | >5 |  |
| Duration (days) | 1 | 2 | | | >3 |  |
| Temperature (°C) | 37.1-38.4 | 38.5-38.9 | | | >39.0 |  |
| Dehydration | N/A | 1-5% or some | | | >6% or severe | . |
| Treatment | Rehydration | Hospitalization | | | N/A | Participants who are “hospitalized” for at least 24 hours OR who receive IV therapy are considered “hospitalized” and receive a corresponding score of 2 points for this parameter |
|  | **VIDA Modified Vesikari Score (mVS)** | | | | |  |
| Diarrhea |  | |  |  | |  |
| Max. no. stools/day | 3 | | 4-5 | >6 | | 1-2 days not permissible; definition of diarrhea requires >3 stools/day |
| Duration (days) | 1-4 | | 5 | 6-7 | | Cannot exceed 7; enrolment criteria requires <7 days |
| Vomiting |  | |  |  | |  |
| Max. no. emesis/day | 1 | | 2-4 | >5 | |  |
| Duration (days) | 1 | | 2 | >3 | | May be truncated because of diarrhea duration enrolment criterion |
| Temperature (°C) | 37.1-38.4 | | 38.5-38.9 | >39.0 | | No change |
| Dehydration | N/A | | some | severe | | No change |
| Treatment | Rehydration | | Hospitalization/IV | N/A | | No change |

| **Table 2. Risk factors for development of persistent diarrhea among 4,606 children with moderate-to-severe diarrhea in GEMS** | | | | |
| --- | --- | --- | --- | --- |
|  | | N | N (%) persistent diarrhea | P |
| **GEMS** | | 4535 | 569 (12.55) |  |
| **Demographic features** | | | | |
| Age stratum | 0-11 months | 1799 | 254 (14.1) | **0.02** |
|  | 12-23 months | 1546 | 188 (12.2) |  |
|  | 24-59 months | 1190 | 127 (10.7) |  |
| Study site | Kenya | 1473 | 304 (20.6) | **<0.0001** |
|  | Mali | 2033 | 210 (10.3) |  |
|  | The Gambia | 1029 | 55 (5.3) |  |
| **Socioeconomic indicators** | | | | |
| Household children | > 2 children < 5 years | 2087 | 197 (9.4) | **<0.0001** |
|  | <2 children < 5 years | 2448 | 372 (15.2) |  |
| Caretaker education | Less than primary school | 3360 | 363 (10.8) | **<0.0001** |
|  | At least primary school | 1175 | 206 (17.5) |  |
| Electricity in home | No | 2403 | 360 (15.0) | **<0.0001** |
|  | Yes | 2132 | 209 (9.8) |  |
| **Clinical findings** | | | | |
| Diarrhea type | Watery Diarrhea | 3970 | 486 (12.2) | 0.10 |
|  | Bloody Diarrhea | 565 | 83 (14.7) |  |
| Stunted | No | 3457 | 428 (12.4) | 0.54 |
|  | Yes | 1078 | 141 (13.1) |  |
| Fever | No | 3478 | 441 (12.7) | 0.63 |
|  | Yes | 1056 | 128 (12.1) |  |
| Vomiting | No | 2591 | 336 (13.0) | 0.33 |
|  | Yes | 1943 | 233 (12.0) |  |
| No. diarrhea stools/day | <5 | 3835 | 478 (12.5) | 0.69 |
|  | >5 | 700 | 91 (13.0) |  |
| WHO-defined dehydration | None | 428 | 49 (11.5) | 0.06 |
|  | Some | 3311 | 400 (12.1) |  |
|  | Severe | 796 | 120 (15.1) |  |
| Lethargy | No | 2451 | 326 (13.3) | 0.10 |
|  | Yes | 2082 | 243 (11.7) |  |
| Treated with WHO recommended antibiotics | No | 4234 | 540 (12.8) | 0.11 |
|  | Yes | 301 | 29 (9.6) |  |

| Table 3. Treatment with WHO recommended antibiotics among patients with moderate to severe diarrhea from VIDA study | | | | |
| --- | --- | --- | --- | --- |
| Characteristic |  | N | N (%) treated w/ WHO recommended antibiotics | P-value^1^ |
|  |  |  |  |  |
| Total Population |  | 4560 | 882 (19.3) |  |
| Children with Bloody Diarrhea | | 697 | 434 (62.3) |  |
| Dysentery duration | Acute | 631 | 397 (62.9) | 0.27 |
|  | Persistent | 66 | 37 (56.1) |  |
| Age Group | 0-11 m | 169 | 92 (54.4) | **0.04** |
|  | 12-23 m | 267 | 178 (66.7) |  |
|  | 24-59 m | 261 | 164 (62.8) |  |
| Site | Kenya | 197 | 69 (35) | **<0.0001** |
|  | Mali | 59 | 7 (11.9) |  |
|  | The Gambia | 441 | 358 (81.2) |  |
| *Shigella* detected by PCR | No *Shigella* | 233 | 119 (51.1) | **<0.0001** |
|  | *Shigella* | 464 | 315 (67.9) |  |
| Case attributed to *Shigella*^3^ | No *Shigella* | 306 | 163 (53.3) | **<0.0001** |
|  | *Shigella* | 391 | 271 (69.3) |  |
| Enrollment HAZ | Stunted | 145 | 91 (62.8) | 0.89 |
|  | Normal | 552 | 343 (62.1) |  |
| Children with Watery Diarrhea | | 3863 | 448 (11.6) |  |
| Diarrhea duration | Acute | 3493 | 420 (12.0) | **0.01** |
|  | Persistent | 370 | 28 (7.6) |  |
| Age Group | 0-11 m | 1452 | 180 (12.4) | 0.18 |
|  | 12-23 m | 1332 | 137 (10.3) |  |
|  | 24-59 m | 1079 | 131 (12.1) |  |
| Site | Kenya | 1279 | 18 (1.4) | **<0.0001** |
|  | Mali | 1486 | 139 (9.4) |  |
|  | The Gambia | 1098 | 291 (26.5) |  |
| *Shigella* detected by PCR | No *Shigella* | 1116 | 150 (13.4) | 0.02 |
|  | *Shigella* | 2747 | 298 (10.9) |  |
| Case attributed to *Shigella*^3^ | No *Shigella* | 3354 | 375 (11.2) | 0.04 |
|  | *Shigella* | 509 | 73 (14.3) |  |
| Enrollment HAZ | Stunted | 865 | 103 (11.9) | 0.75 |
|  | Normal | 2998 | 345 (11.5) |  |

**Table 4. Treatment with zinc and persistent diarrhea by age and site**

| Age stratum | Total N | Zinc rx, N (%) | Developed PD, N | Zinc rx, N (%) | P-value* |
| --- | --- | --- | --- | --- | --- |
|  |  | Kenya |  |  |  |
| 0-11 mo | 559 | 532 (95.2) | 104 | 101 (97.1) |  |
| 12-23 mo | 504 | 494 (98.0 | 77 | 75 (97.4) |  |
| 24-59 mo | 420 | 407 (96.9) | 48 | 48 (100) |  |
| Total | 1483 | 1433 (96.7) | 229 | 224 (97.8) | 0.27 |
|  |  | Mali |  |  |  |
| 0-11 mo | 564 | 47 (8.3) | 30 | 3 (10.0) |  |
| 12-23 mo | 533 | 29 (5.4) | 26 | 1 (3.8) |  |
| 24-59 mo | 449 | 12 (3.7 | 10 | 1 (10.0) |  |
| Total | 1546 | 88 (5.7) | 66 | 5 (7.6) | 0.50 |
|  |  | The Gambia |  |  |  |
| 0-11 mo | 506 | 231 (45.7) | 50 | 17 (34.0) |  |
| 12-23 mo | 576 | 292 (50.7) | 51 | 23 (45.1) |  |
| 24-59 mo | 486 | 237 (48.8) | 40 | 14 (35.0) |  |
| Total | 1568 | 760 (48.5) | 141 | 54 (38.3) | 0.01 |

*P values were calculated for risk of persistent diarrhea among children who received zinc compared to those who did not by site

**Figure 1.  Memory aid for caretakers to record the presence of diarrhea for 14 days after enrollment.**

**
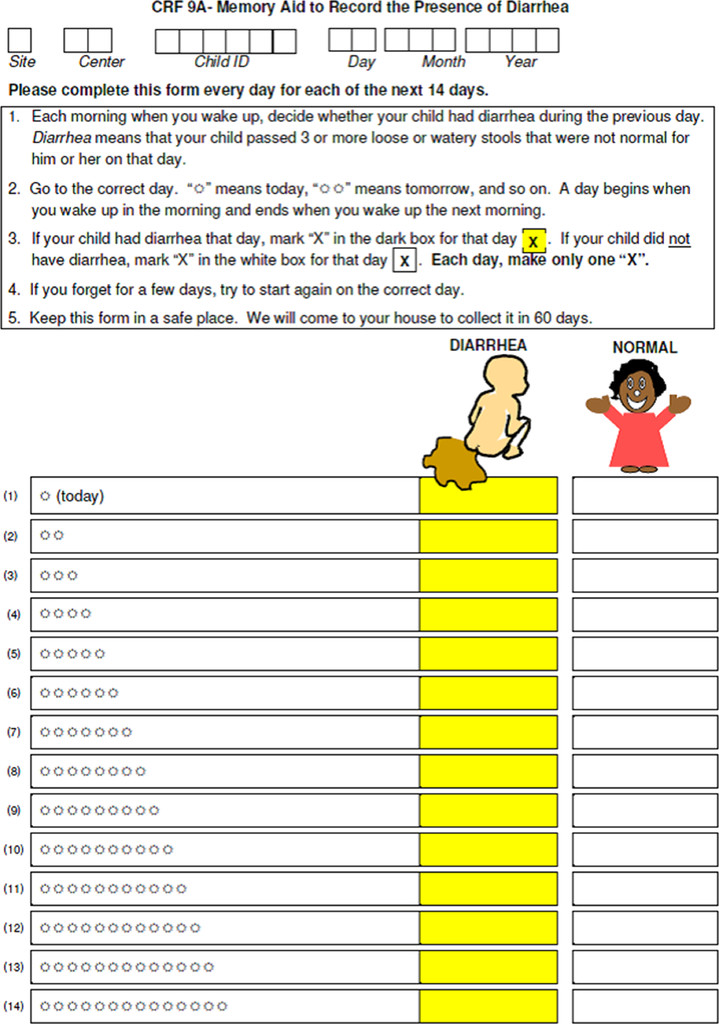
**

**Figure 2. Odds ratios and 95% confidence intervals for rotavirus and enteroaggregative *E. coli* by site.**

**
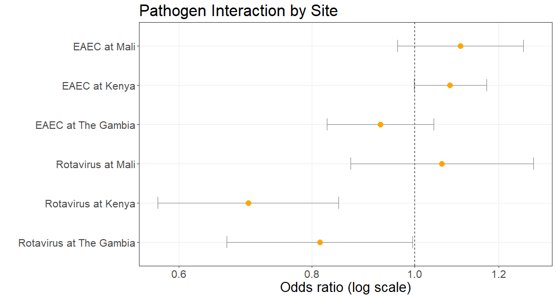
**
